# Estimating the Transmissibility of Mumps: A Modelling Study in Wuhan City, China

**DOI:** 10.3389/fmed.2021.683720

**Published:** 2021-08-03

**Authors:** Ying Peng, Tianlong Yang, Yuanzhao Zhu, Qingqing Hu, Yao Wang, Zeyu Zhao, Jia Rui, Shengnan Lin, Xingchun Liu, Jingwen Xu, Meng Yang, Bin Deng, Jiefeng Huang, Weikang Liu, Li Luo, Chan Liu, Zhuoyang Li, Peihua Li, Deguang Kong, Xiaobing Yang, Tianmu Chen

**Affiliations:** ^1^Wuhan Centers for Disease Control and Prevention, Wuhan, China; ^2^State Key Laboratory of Molecular Vaccinology and Molecular Diagnostics, School of Public Health, Xiamen University, Xiamen, China; ^3^Division of Public Health, School of Medicine, University of Utah, Salt Lake City, UT, United States

**Keywords:** China, effective reproduction number, mathematical model, mumps, transmissibility, Wuhan

## Abstract

Despite the adoption of a national immunization program in China, the incidence of mumps remains high. This study aimed to describe the epidemiological characteristics, including the time, region, occupation, and age, of mumps in Wuhan from 2005 to 2018 and to evaluate its transmissibility. In this study, the susceptible–exposed–infectious–asymptomatic–recovered (SEIAR) model fitted the actual incidence data of mumps. The effective reproduction number (*R*_*t*_) was used to evaluate and compare the transmission capacity in different areas. From 2005 to 2018, there were 36,415 cases. The incidence of mumps was highest among people aged 5–10 years (460.02 per 100,000). The SEIAR model fitted the reported mumps data well (*P* < 0.01). The median transmissibility (*R*_*t*_) was 1.04 (range = 0–2.50). There were two peak spreads every year (from March to May and from October to December). The *R*_*t*_ peak always appeared in the first 2 months of the peak incidence rate. The peak time of the epidemic spread of mumps was 1–2 months earlier than the peak incidence rate. The prevention and control measures of vaccination for children aged 5–10 years should be taken before the peak transmission capacity each year, 2 months before the peak of the outbreak, to reduce the spread of mumps.

## Introduction

Mumps is an acute respiratory infectious disease caused by the mumps virus, *Rubulavirus* (1). It occurs mainly in children and adolescents and is highly transmissible (2). The main symptoms include fever, muscle pain, and headache. Pain and swelling of one or both parotid and salivary glands soon follow the presentation of the main symptoms. The mumps virus is a member of the Paramyxoviridae RNA family of viruses (3). It is an envelope RNA virus that is transmitted through the saliva, respiratory secretions, or close contact with an infected person (4). Up to 10% of patients with mumps develop encephalitis, which can lead to death or disability (5).

Mumps is usually mild and self-limiting, but its burden should not be underestimated (6). Mumps is an epidemic viral disease worldwide. More than 90% of cases are not reported (7). From 2005 to 2010, an average of 560,000 cases were reported worldwide (8). In countries such as Egypt, where the population is not provided with routine mumps vaccination, the burden of mumps is very high (100–1,000 cases per 100,000 people), and the epidemic peak occurs every 2–5 years (5, 9). In several developed countries, the outbreak of mumps is cyclical because of vaccination being voluntary (2). Even in countries where mumps vaccines are used through national immunization programs, mumps demonstrates a recurrent trend (10–12). In the United States, for instance, mumps outbreaks occur in highly immunized school-age populations (13–15). In some countries, such as Iraq, the incidence rate of mumps has been increasing due to long-term military conflicts, economic sanctions, and wars (16).

In China, mumps is still an important public health problem. The incidence rate of mumps in Mainland China remains high. Since mumps surveillance was first conducted in 2004, the average annual incidence rate of mumps has been 24 cases per 100,000 people (17). Although the mumps vaccine has been used in the national vaccination program for children aged 18–24 months since 2007, the incidence of mumps has not decreased significantly between 2008 and 2010. In fact, the incidence of mumps was slightly higher from 2008 to 2010 than that from 2004 to 2007 (8).

Children and adolescents are particularly vulnerable to mumps. In a prospective study (18) on the effect of a single dose of the measles, mumps, and rubella (MMR) vaccine on children's immunity against mumps (18), it was reported that children in kindergartens and elementary schools who only received a single dose of MMR had a high risk of mumps infection, and their immunity gradually declined over time. In recent years, mumps still poses a considerable disease burden, and its incidence is uneven in China. The reported incidences of mumps in southern provinces, such as Guangdong, rank among the top three for China. Furthermore, the incidence rates of mumps in western provinces are higher than those in eastern and central provinces (8, 19). The incidence rates of mumps in most parts of China have been affected by precipitation, air pressure, temperature, and wind speed. The incidence of mumps in North and Southwest China is more susceptible to climatic factors (20).

Mathematical models are important tools for understanding the spread and control of infectious diseases and implement a number of practical prevention and control measures to curb the spread of an epidemic. Several studies have used epidemic dynamics models to analyze mumps. In one study on the seasonal model of mumps in China, a non-autonomous susceptible–vaccinated–exposed–mildly infectious–severely infectious–hospitalized–recovered model (21, 22), which described the seasonal variation of the transmission rate of mumps, was proposed (21, 22). One study established a state space model to describe the transmission process and the trend of mumps before and after the implementation of the vaccine policy (23). However, this study considered only the effect of vaccine prevention and control and did not consider the influence of the infectious source, transmission route, and the susceptible population on the prevention and control of infectious diseases (23). Moreover, it did not introduce the recessive infection into the dynamic model, so it had certain limitations. In recent years, in addition to ordinary differential equations, studies have focused on the establishment of the seasonal autoregressive integrated moving average model to fit the epidemic trend of mumps (24). However, these models failed to clarify the process of disease transmission and could not quantitatively evaluate disease transmissibility.

Only a few studies have used the infectious disease dynamics model to simulate and predict the incidence trend of mumps. Therefore, we established an ordinary differential equation of mumps, namely, the susceptible–exposed–infectious–asymptomatic–recovered (SEIAR) model to describe the epidemic trend of mumps in Wuhan from 2005 to 2018 and to evaluate its transmissibility.

## Materials and Methods

### Study Design

In this study, a mathematical epidemiological method was used to establish the SEIAR model with seasonally adjusted parameters to mine data in order to determine the characteristics and transmissibility of mumps.

### Data Collection

The research data on mumps cases from January 1, 2005 to December 31, 2018, in Wuhan City were obtained from the Chinese Information System for Disease Control and Prevention (CISDCP). As a class C notifiable communicable disease, mumps cases should be reported through the CISDCP within 24 h of diagnosis. The diagnosis of mumps was made by following the criteria for mumps set forth by the National Health Commission of the People's Republic of China. Information on sex, age, occupation, address, illness onset date, and date of diagnosis was included in the data. The population data were obtained from the Wuhan Statistical Yearbook.

### Model Building

In the SEIAR model, the population was divided into five categories: susceptible (*S*), exposed (*E*), infectious (*I*), asymptomatic (*A*), and recovered/removed (*R*). The model was based on the following assumptions: (1) *n* is the total population, μ is the birth rate, and *m* is the natural death rate; (2) the infection rate coefficient after effective contact between *S* and *I* is β, and that asymptomatic *A* is infectious and the transmissibility is *k* (0 ≤ *k* ≤ 1) times that of infectious *I*; then, at moment *t*, the number of new infections is β*S* (*I* + *kA*); (3) the proportion of asymptomatically infected is *p*, and the exposed individuals become symptomatic and asymptomatic cases after an incubation period (1/ω) and a latent period (1/ω′); the numbers of people who change from *E* to *A* and *I* at time *t* are *p*ω′*E* and (1 – *p*)ω*E*, respectively; the model assumes that the incubation period is equal to the latent period; and (4) symptomatic and asymptomatic patients are defined as removed persons after an infectious period of 1/γ and 1/γ′, respectively.

**Figure 1:**
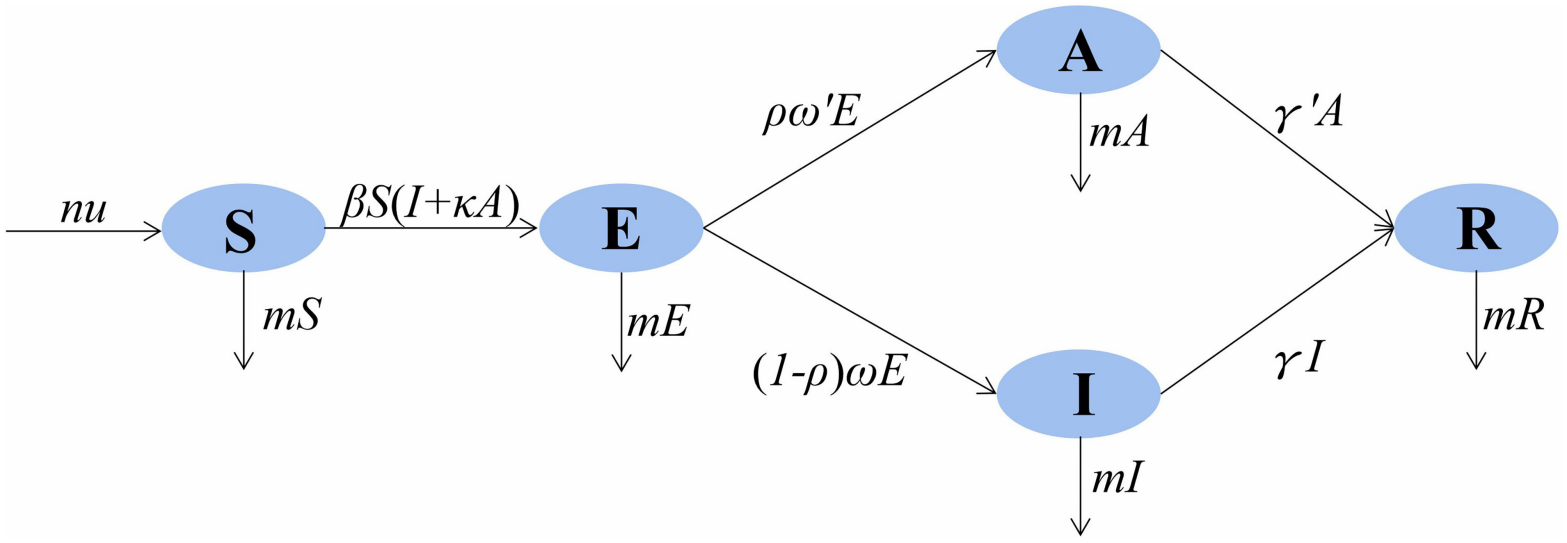
SEIAR (susceptible–exposed–infectious–asymptomatic–recovered) model framework of mumps in Wuhan City.

The model framework is shown in Figure 1. The equations of the model are as follows:

$$\begin{array}{l} d\left(S\right)/dt\;=\;n\mu -\beta S\left(I\;+\;kA\right)\;-\;mS\\ d\left(E\right)/dt\;=\;\beta S\left(I\;+\;kA\right)\;-\;\rho \omega^{\prime }E\;-\;\left(1\;-\;\rho \right)\omega E\;-\;mE\\ d\left(I\right)\;/dt\;=\;\left(1\;-\;\rho \right)\omega E\;-\;\gamma I\;-\;mI\\ d\left(A\right)/dt\;=\;\rho \omega^{\prime }E\;-\;\gamma^{\prime }A\;-\;mA\\ d\left(R\right)/dt\;=\;\gamma I\;+\;\gamma^{\prime }A\;-\;mR\\ \;n\;=\;S\;+\;E\;+\;I\;+\;A\;+\;R \end{array}$$

### Seasonality of Transmission

In this study, the seasonality of transmission was considered. According to the mechanism of the SEIAR model we established, the seasonality should be dynamic, focusing on β. Therefore, a trigonometric function was adopted as follows:

$$\begin{array}{l} \beta =\beta_{0}\left[1+\sin\left(\frac{2\pi \left(t+\alpha \right)}{T}\right)\;\right] \end{array}$$

In the equation, β_0_, *t*, α, and *T* refer to the baseline of the transmission relative rate, time, a constant adjusting the position of time, and the time span of the season cycle respectively.

### Parameter Estimation

According to the model, there are six parameters involved in the SEIAR model of mumps: birth rate μ, natural mortality rate *m*, infection coefficient β, proportion of the asymptomatic ρ, incubation relative rate ω, latent relative rate ω′, and the recovery removal rate coefficient γ. There are six variables involved, namely, the total population *N*, susceptible *S*, latent period *E*, dominant infection *I*, recessive infection *A*, and emigrant *R*. The average incubation period of mumps is 18 days. This study, with reference to previous studies and combined with actual conditions in Wuhan, set the incubation period in the range between 10 and 20 days. This value was 12 days, so ω = 1/12. The value was 0.08333 (25). With reference to the research on the definition and selection of disease course according to the actual epidemic situation combined with the actual epidemic situation in Wuhan, the disease course range was set to 5–25 days, with a value of 17 days and γ = 1/17. The value was 0.05882 (26). The significance and estimation of each parameter and the initial value setting of the variables are shown in Table 1.

**Table 1:** Description and source of parameters in the SEIAR model.

**Parameter**	**Description**	**Method**	**Unit**	**Range**	**Value**
μ	Birth rate	Reference	day^−1^	0–1	–
*m*	Mortality rate	Reference	day^−1^	0–1	–
β	Transmission relative rate	Curve fitting	–	0–1	–
ρ	Proportion of those asymptomatic	Reference	1	0.15–0.27	0.2
ω	Incubation relative rate	Reference	day^−1^	0.05–0.1	0.08333
ω′	Latent relative rate	Reference	day–^1^	0.05–0.1	0.08333
γ	Removed rate of those infectious	Reference	day^−1^	–	0.05882
γ′	Removed rate of those asymptomatic	Reference	day^−1^	–	0.05882

*SEIAR, susceptible–exposed–infectious–asymptomatic–recovered*.

### Quantitative Evaluation of Transmissibility

The transmissibility of the mumps virus was quantified using the basic reproduction number (*R*_0_) (27). *R*_0_ refers to the number of new cases that could arise from direct transmission of one infectious source in the susceptible population during its infection period. When *R*_0_ > 1, the disease will spread and cause an epidemic; when *R*_0_ < 1, the epidemic terminates; when *R*_0_ = 1, the disease will neither cause an epidemic nor stop. When the intervention measures are taken, *R*_0_ reflects the real transmissibility of infectious diseases. It is expressed as the effective reproduction number (*R*_*t*_) (28, 29). The effectiveness of the prevention and control measures can be evaluated by calculating the value of *R*_*t*_ in different time periods and comparing the value with the threshold of 1 to the changes in the different time periods. The formula for *R*_*t*_ in the SEIAR model is as follows:

$$\begin{array}{l} R_{t}=\beta S\left(\frac{1-\rho }{\gamma }+\frac{\kappa \rho }{\gamma \prime }\right) \end{array}$$

### Global Spatial Autocorrelation Analysis

The OpenGeoDa 1.2.0 software was used to calculate the global Moran's *I* coefficient and to detect the overall spatial autocorrelation. The range of Moran's *I* coefficient is [−1,1]. When the value is >0, the spatial correlation is positive, and the larger the value, the stronger the spatial clustering. When the value is <0, the spatial correlation is negative. The closer the value is to −1, the greater the spatial difference; the space is irrelevant. Of note is that the significance of Moran's *I* coefficient is based on the *Z* value and the *P*-value.

### Simulation Method and Statistical Analyses

We used the Berkeley Madonna 8.3.18 software (developed by Robert Macey and George Oster of the University of California at Berkeley; Copyright© 1993–2001 Robert I. Macey and George F. Oster) to model the actual situation of mumps in Wuhan. The simulation method (Runge–Kutta fourth-order method, with tolerance set to 0.001) was the same as those used in previously published studies. The model was used to fit the data to calculate the parameters. Transmissibility was calculated according to the parameters. The goodness of fit of mumps in Wuhan was calculated using the coefficient of determination (*R*^2^) values and the *P*-values in SPSS 21.0 (IBM Corp, Armonk, NY, USA).

## Results

### Epidemiological Description of Mumps in Wuhan

**Figure 2:**
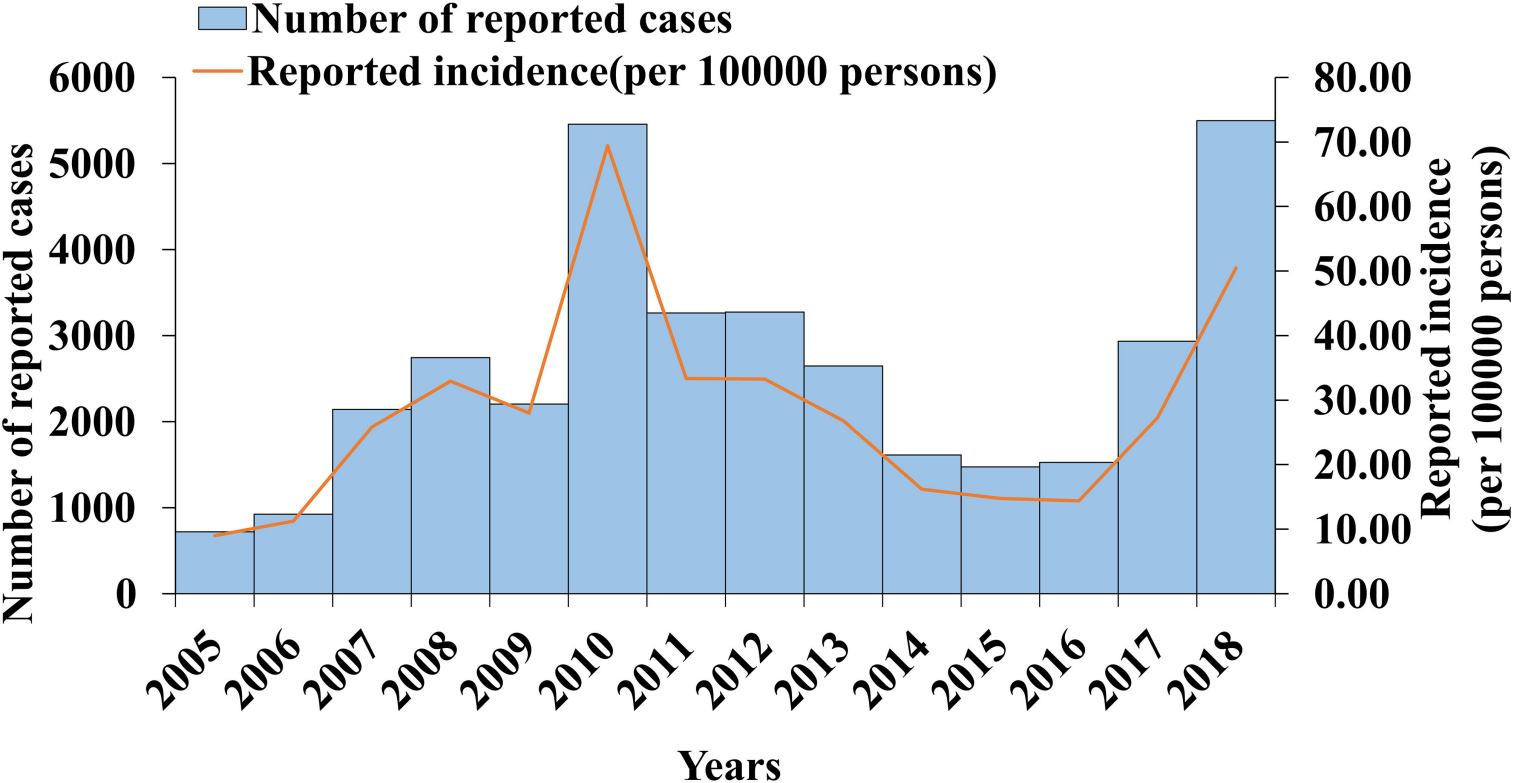
Mumps cases and incidence rate in Wuhan City in 2005–2018.

From 2005 to 2018, 36,415 cases of mumps were reported in Wuhan, with a minimum incidence rate of 8.98 per 100,000 in 2005 and a maximum of 69.72 per 100,000 in 2010. From 2005 to 2006, the incidence rate of mumps increased gradually. The first large increase occurred between 2006 and 2008. The incidence rate decreased slightly in 2009, but there was a second significant increase that reached the peak in 2010 (69.42 per 100,000). The incidence rate dropped sharply from 2010 to 2014, decreased slowly in 2016, and increased again from 2016 to 2018. The results are shown in Figure 2.

**Figure 3:**
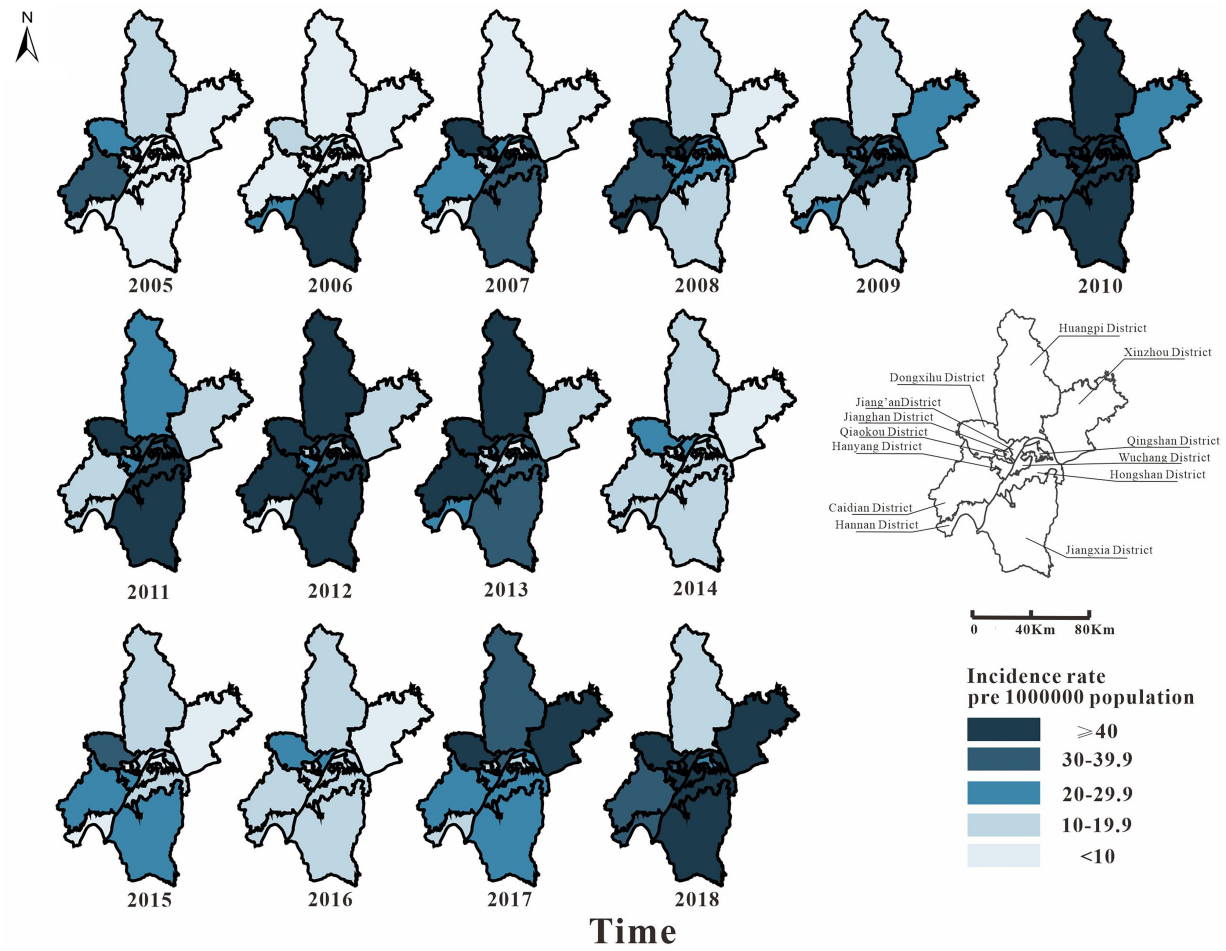
Map of the incidence rate of mumps in Wuhan City in 2005–2018. The map depicted in this figure was taken from Wikimedia Commons (http://commons.wikimedia.org/wiki/Main_Page).

From 2005 to 2018, 36,355 cases of mumps were reported in 13 districts of Wuhan. Among them, Hongshan District (5,205 cases), Jiang'an District (4,543 cases), and Wuchang District (4,280 cases) had more cases. From 2005 to 2010, the epidemic area was transferred from the surrounding area to the middle area. In 2010, the incidence rate of mumps in Wuhan was high (69.72 per 100,000). From 2010 to 2016, the incidence rate in the middle area dropped significantly; it began to decline in the surrounding area later than it did in the middle area. The number of the surrounding areas began to decrease significantly from 2014. The incidence rate of each district has been increasing since 2016. In general, the incidence rates in the districts of Caidian, Dongxihu, Hongshan, and Jiang'an were relatively high, while those of Hanyang, Qingshan, and Xinzhou were low (Figure 3).

**Figure 4:**
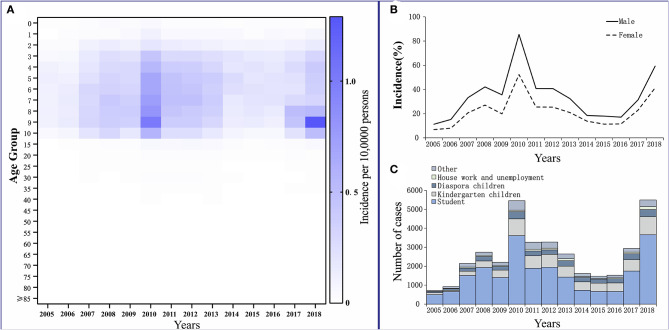
Distribution of mumps in Wuhan City. **(A)** Age distribution. **(B)** Gender distribution. **(C)** Occupation distribution.

The reported cases of mumps involved mainly patients who were 3–15 years old. They accounted for 79.24% of the total number of cases. Among them, patients who were 5–10 years old had the highest average annual incidence rate of 460.02 per 100,000. Patients aged ≥40 years had the lowest reported incidence, which accounted for only 3.12% of the total number of cases, and the average annual incidence for this age group was 30.37 per 100,000 (Figure 4A).

**Figure 5:**
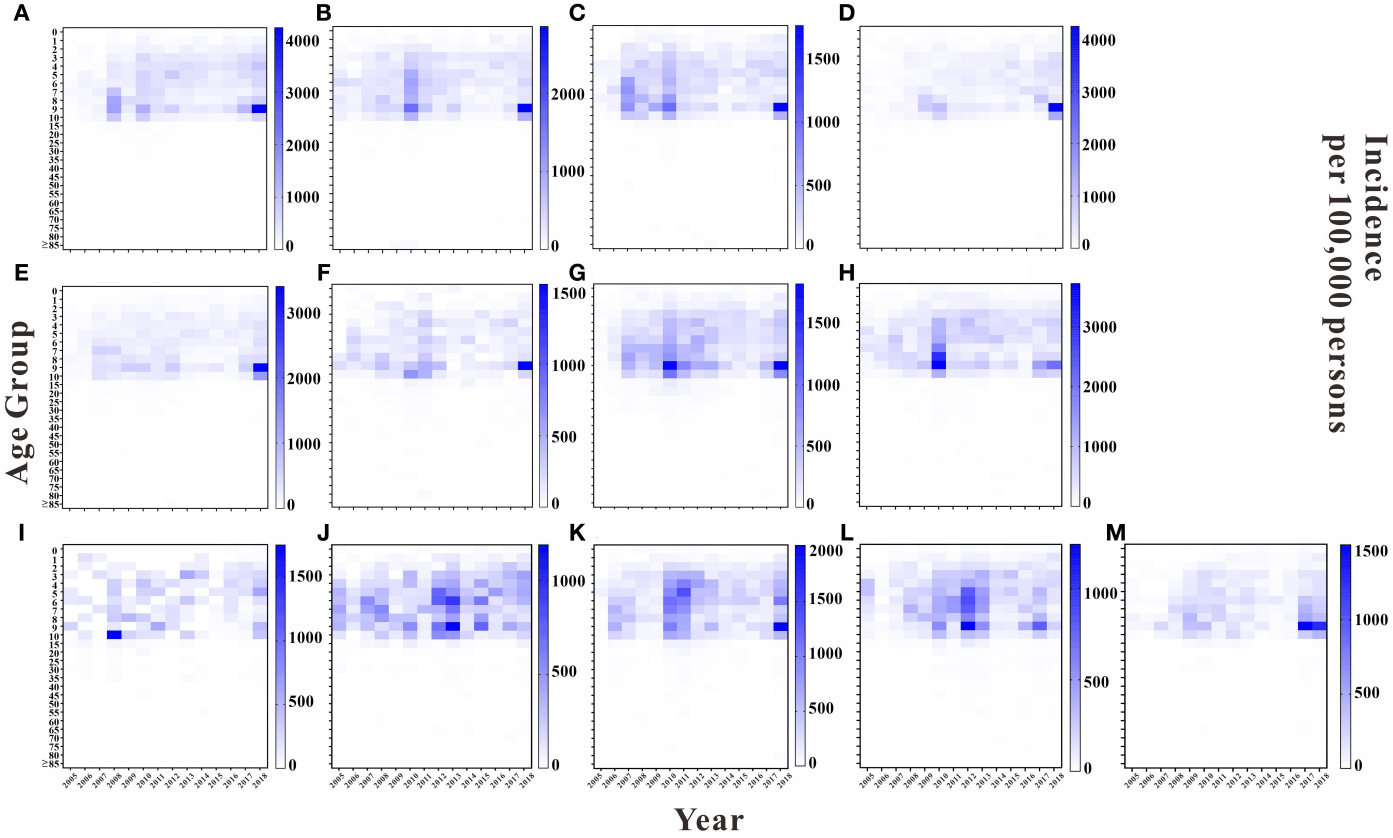
Incidence rate of each age group in Wuhan City by region in each year. **(A)** Jiangan District. **(B)** Jianghan District. **(C)** Qiaokou District. **(D)** Hanyang District. **(E)** Wuchang District. **(F)** Qingshan District. **(G)** Hongshan District. **(H)** Dongxihu District. **(I)** Hannan District. **(J)** Caidian District. **(K)** Jiangxia District. **(L)** Huangpi District. **(M)** Xinzhou District.

We analyzed the incidence rate for each age group in each year in 13 regions of Wuhan. Overall, the highest average annual incidence rate was found in the 5 to 10-year-old age group in each region in each year. The years 2010 and 2018 had higher average annual incidence rates in this age group in each region than did the other years. Except for Hannan District and Jiangxia District, the average annual incidence rate of the 9 to 10-year-old age group was higher in the remaining regions than that in the other age groups, with the top three regions being East and West Lake District, Jiang'an District, and Hanyang District, with average annual incidence rates of 882.61, 834.97, and 628.19 per 100,000, respectively. The results are shown in Figure 5.

Among the reported cases of mumps in Wuhan from 2005 to 2018, 22,641 cases were males, with an average annual reported incidence rate of 34.14 per 100,000. There were 13,774 cases among females, with an average annual reported incidence rate of 21.96 per 100,000. The incidence of cases in males was higher than that in females. The ratio of the incidence in males to females was 1.55:1 (Figure 4B).

Students accounted for the highest proportion in the total number of cases, at 61.33% (22,333 cases). They also accounted for more than 50% of the total affected population every year, except in 2014–2016. For kindergarten and scattered children, the cumulative numbers of cases for 14 years were 6,826 and 3,235, accounting for 18.75 and 8.88% of the total number of cases, respectively (Figure 4C).

### Model Fitting Results and Effect Evaluation

**Figure 6:**
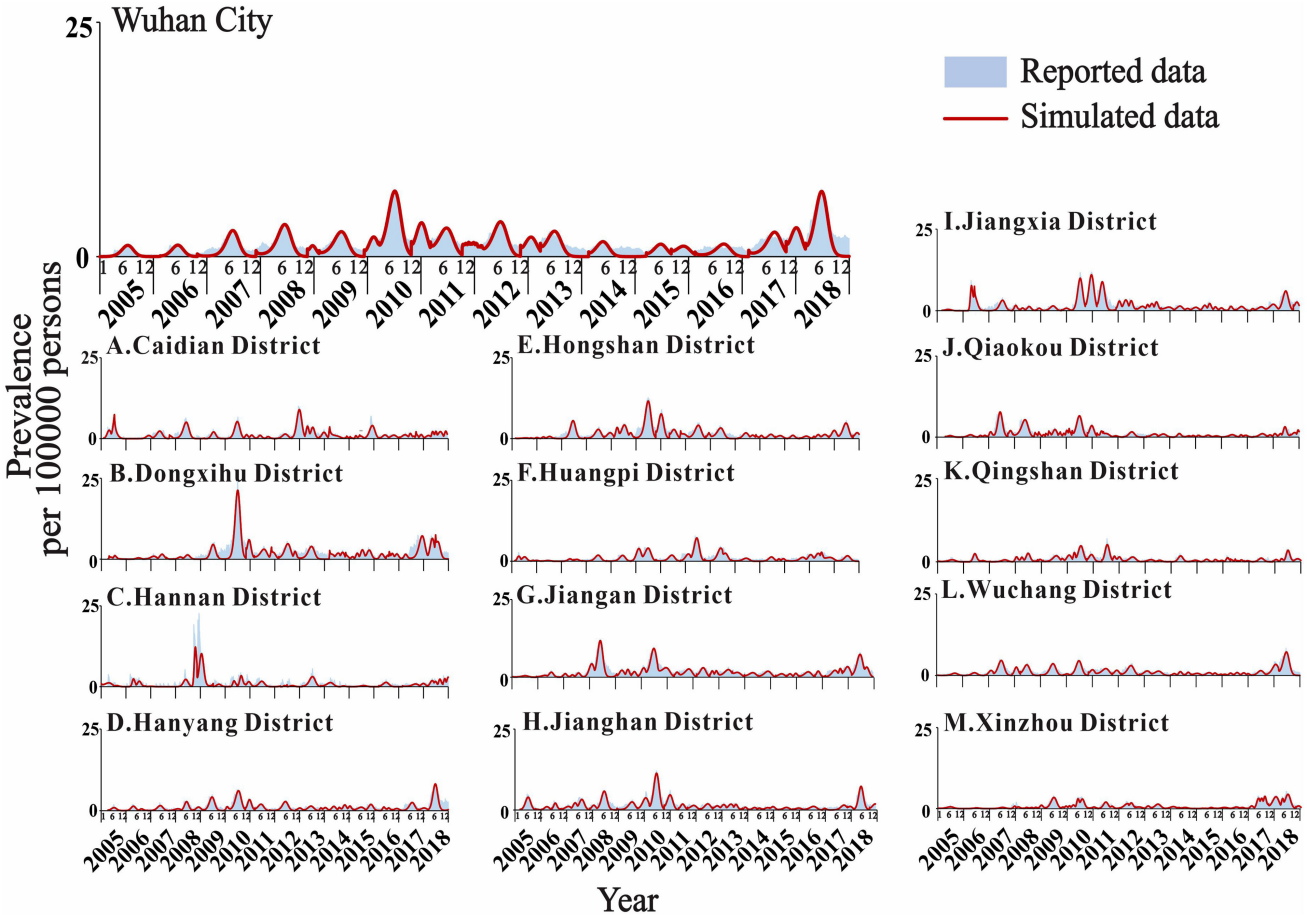
Fitting results of mumps cases in Wuhan City and the 13 districts.

The actual epidemic situation of mumps in Wuhan and its 13 districts from 2005 to 2018 was fitted (Figure 6). The results showed that there were reported cases in Wuhan every month with seasonal high incidence characteristics. The incidence started to rise in February each year, reached a peak in April and July, fell back, reached its first trough in September, increased slowly, peaked for a second time from November to January of the following year, and reached its second trough in February. The overall incidence showed two peaks and two troughs, one large and one small. Among them, the incidence in June 2010 reached the highest peak in history. Among the 13 districts in Wuhan, Xinzhou had the best-fitting effect (*R*^2^ = 0.922, *P* < 0.001). The determination coefficients of the 13 districts ranged from 0.576 to 0.922, all of which were statistically significant (*P* < 0.001) (Table 2).

**Table 2:** Goodness-of-fit test for Wuhan City and its 13 districts.

**Area**	***R*^**2**^**	***P*-value**
Wuhan City	0.878	<0.001
Caidian District	0.904	<0.001
Dongxihu District	0.881	<0.001
Hannan District	0.576	<0.001
Hanyang District	0.802	<0.001
Hongshan District	0.902	<0.001
Huangpi District	0.883	<0.001
Jiangan District	0.886	<0.001
Jianghan District	0.907	<0.001
Jiangxia District	0.901	<0.001
Qiaokou District	0.877	<0.001
Qingshan District	0.876	<0.001
Wuchang District	0.865	<0.001
Xinzhou District	0.922	<0.001

### Transmissibility Calculation Results

**Figure 7:**
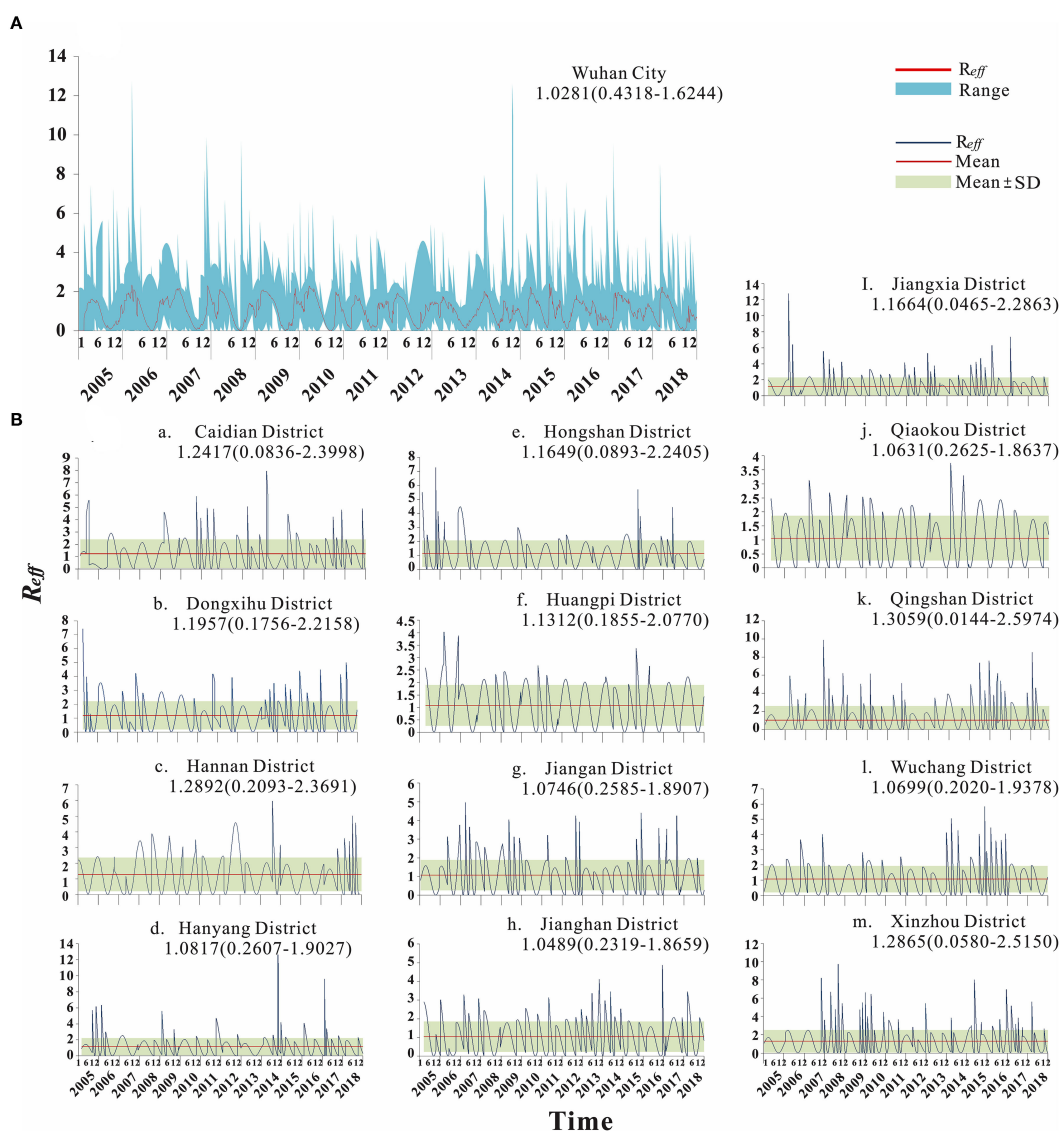
Calculation results of transmissibility of Wuhan City and 13 districts. **(A)** Transmissibility of mumps in Wuhan City. **(B)** Transmissibility of mumps in 13 districts of Wuhan.

**Figure 8:**
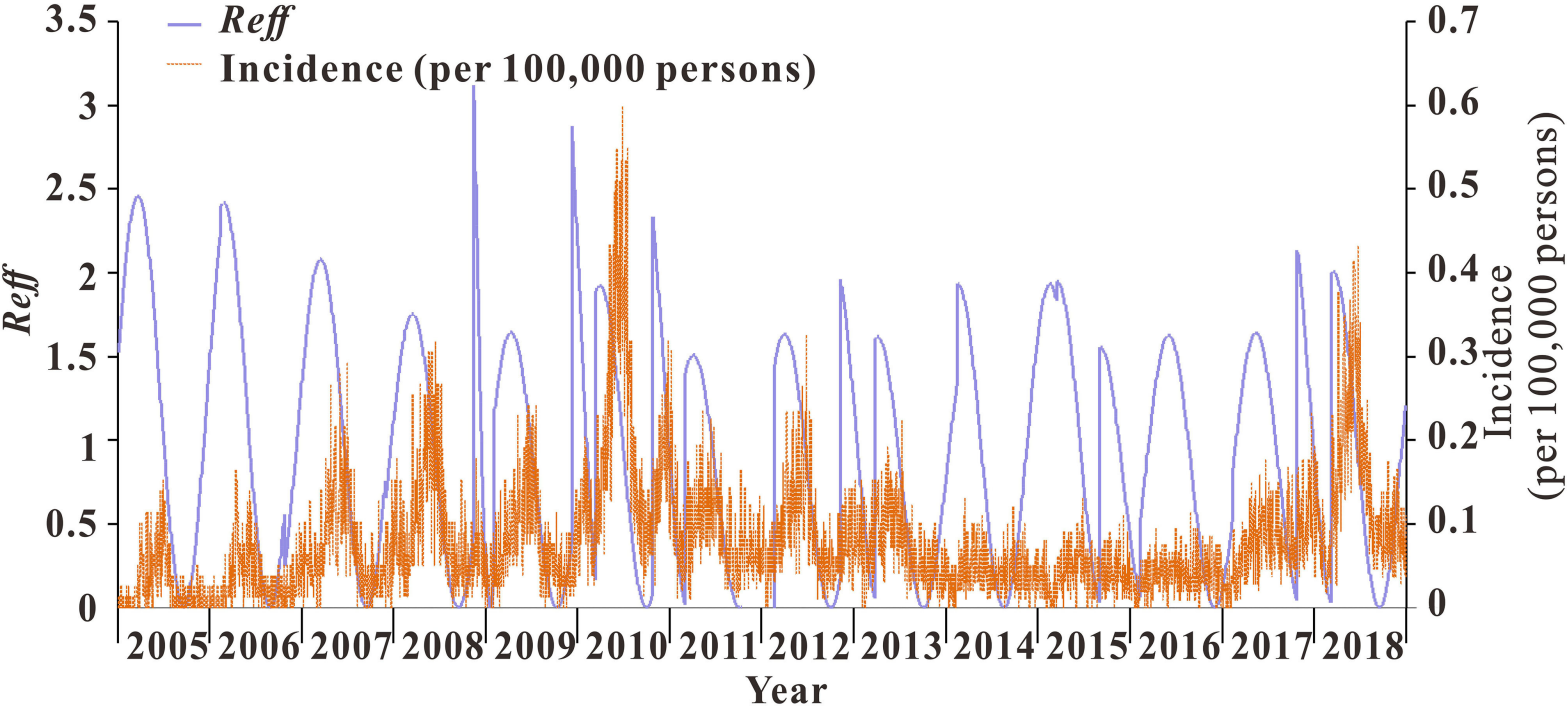
Trend of incidence rates and transmissibility of mumps in Wuhan City.

From 2005 to 2018, the median transmissibility of mumps in Wuhan was 1.04 (range = 0–2.50), showing obvious seasonal characteristics. Each year, there were two transmission peaks (from March to May and from October to December). The calculation results of the transmissibility of the 13 districts in Wuhan are shown in Figure 7. The trend of *R*_*t*_ in Wuhan was consistent with the incidence. The peak of *R*_*t*_ always appeared in the first 1–2 months of the peak of incidence. There were seasonal and two peaks, respectively (Figure 8).

### Spatial Autocorrelation Analysis Results

There was no significant difference in the global Moran's *I* coefficient of mumps in Wuhan from 2015 to 2018 (*P* > 0.05), and the distribution of mumps showed no spatial correlation in the entire region (Table 3).

**Table 3:** Global autocorrelation of the incidence of mumps in Wuhan City from 2005 to 2018.

**Years**	**Moron's *I***	***Z* value**	***P*-value**
2005	−0.149	−0.583	0.330
2006	−0.100	−0.168	0.438
2007	−0.025	0.442	0.284
2008	−0.124	−0.389	0.381
2009	0.013	0.840	0.198
2010	−0.064	0.166	0.407
2011	−0.074	0.078	0.453
2012	−0.112	−0.256	0.452
2013	−0.111	−0.234	0.455
2014	−0.186	−0.934	0.178
2015	−0.091	−0.036	0.469
2016	−0.124	−0.304	0.423
2017	0.018	0.909	0.172
2018	−0.035	0.436	0.307

### Sensitivity Analysis

**Figure 9:**
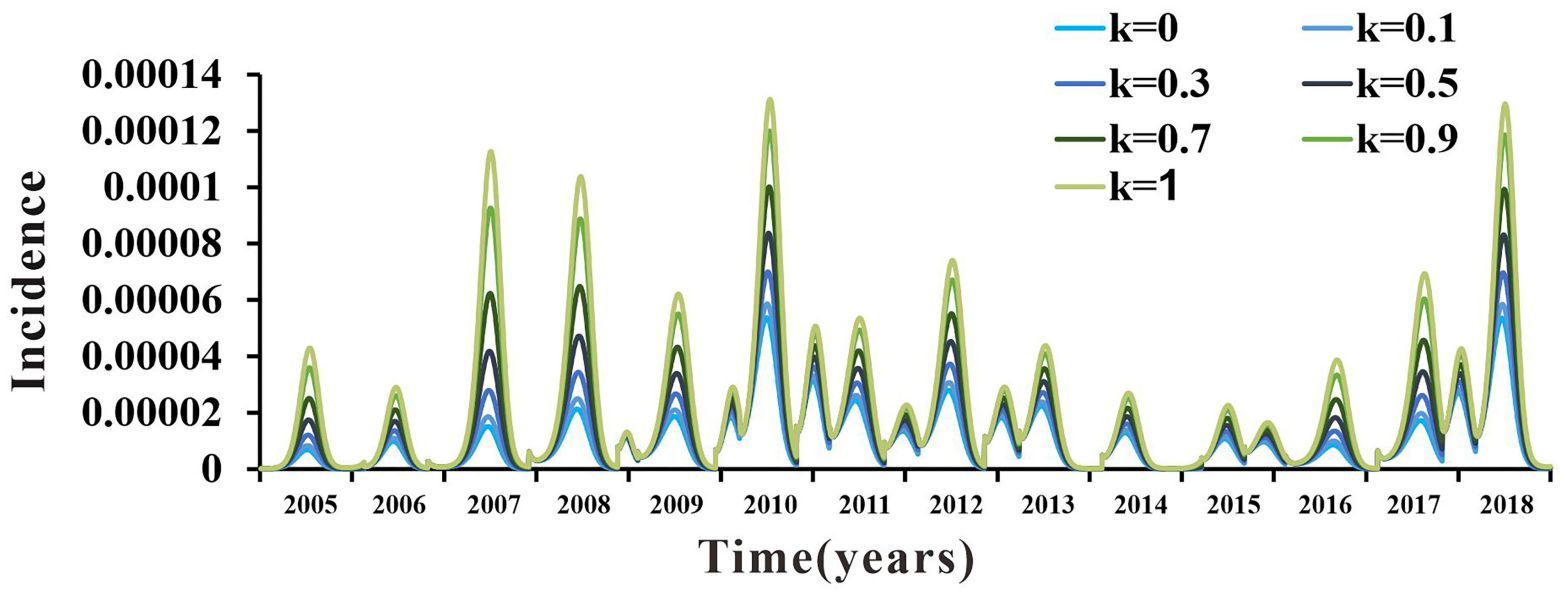
Sensitivity analysis results.

The value of the parameter *k* was still uncertain. There were no clear data or literature to support its value. Therefore, *k* was divided into seven values in the range from 0 to 1 (*k* = 0, *k* = 0.1, *k* = 0.3, *k* = 0.5, *k* = 0.7, *k* = 0.9, and *k* = 1). The coincidence degree of the fitting curves of the seven values of parameter *k* in the range of 0–1 was not high, which indicated that the SEIAR model used in this study was sensitive to it. The results are shown in Figure 9.

## Discussion

In this study, to the best of our knowledge, for the first time, we used a seasonally adjusted SEIAR model to determine the transmissibility of mumps, which could provide guidance in the study of mumps transmissibility and prevention and control measures.

We described the epidemiology of mumps in Wuhan from 2005 to 2018 in terms of incidence trends and the distribution of peak incidence times and populations. Overall, the incidence of mumps in Wuhan has shown a fluctuating trend during this 14-year period. Since 2008, one dose of the MMR vaccine has been administered to children aged 18–24 months. However, prior to 2008, the mumps vaccine was used as a class II vaccine, following the principle of independent, voluntary, and self-funded vaccination. The short duration of the introduction of the MMR vaccine resulted in children aged 5–10 years not receiving the mumps vaccine, being the susceptible population in Wuhan from 2005 to 2010. Thus, despite the introduction of the MMR vaccine in 2008, the incidence of mumps continued to show an upward trend until 2010. In the following years, targeted immunizations were administered in Wuhan to children aged 5–10 years in order to effectively protect the susceptible population, resulting in a downward trend in mumps incidence from 2011 to 2016. Several studies (30, 31) have shown that, after vaccination, the mumps virus-specific antibody concentrations decreased, vaccine effectiveness decreased, and the risk of developing mumps virus infection increased again, which are consistent with the results of our study: the incidence of mumps in Wuhan again showed an increasing trend from 2017 to 2018.

Some studies have confirmed that climatic factors such as temperature and humidity are important factors affecting the incidence of mumps (32, 33). Wuhan is located in the eastern part of Jianghan District and has a subtropical humid monsoon climate. It has been shown that the mumps virus survives longest in the temperature range of 2–4°C; the average winter and spring temperatures in Wuhan are 1–3°C, which provides an impetus for the spread of mumps (34). On combining the incidence map of Wuhan with the results of the global autocorrelation analysis, we found that there was no spatial correlation between the differences in mumps incidence in various districts of Wuhan, implying a random distribution of mumps cases in each district without regional aggregation. In relatively developed areas such as Jianghan District and Dongxihu District, the high incidence may be caused by the high population density, high population mobility, and the high level of diagnosis. In some areas with low socioeconomic status and inadequate medical and health services, such as in the districts of Huangpi, Xinzhou, and Caidian, a high mumps incidence was also observed in some years, which may be due to the untimely mumps vaccination and low coverage (35, 36). Therefore, the actual factors influencing mumps in areas with high incidence should be further studied in addition to the existing immunization policy deficiencies, where the second dose of the mumps vaccine immunization program has not yet been implemented.

Mumps is frequently observed in crowded places such as prisons, kindergartens, boarding schools, military barracks, and others (37). Age, an immunocompromised status, and exposure are risk factors for mumps. According to epidemiological studies of mumps in other regions (38–40), the prevalence was significantly higher in males than in females, which is consistent with our study and may be due to the behavioral patterns of males and females (41, 42), with boys being more active than girls and more likely to have increased exposure to the mumps virus. The present study showed that those under the age of 15 years, especially students in the age group 5–10 years, accounted for the highest proportion of mumps cases. Previous studies have shown that, after one dose of the MMR vaccine in children aged 18–24 months, the antibody positivity rate was only 58.18% after 3 years of immunization up to 4 years of age, and the vaccine positivity rate and protective effect decreased significantly over time (43). This is one of the main reasons why students and children are susceptible to mumps during the school year, along with the weak immunity to the virus in this population. The two peak months for the onset of mumps coincide with the school year, and the confined environment of schools is more conducive to the spread of mumps, so once students are infected, an outbreak of mumps is inevitable. Therefore, it is recommended that schools and kindergartens strengthen the health management of students by strictly checking their vaccination certificates before enrollment and providing health education to avoid potential mumps epidemics.

The infectiousness of mumps varies greatly throughout the year, with abnormal peaks in the winter and spring of each year and a marked increase in the transmission rate, the highest peak being 450,000 times higher than the lowest peak, in a bimodal seasonal distribution, consistently reported in previous studies (44, 45). The present study showed that the frequency of mumps transmission preceded the incidence rate and the peak of transmission preceded the onset of mumps by 1–2 months in Wuhan. This suggests that the increased infectiousness of mumps preceded its outbreak. Therefore, to better control the outbreak, the timing of intervention should be adjusted to 1–2 months before the peak of mumps onset. The spread of the epidemic can be maximized if the correct timing of intervention is chosen before the peak of mumps incidence from March to May and from September to November each year. Currently, the most effective way of controlling the spread of mumps is to increase the mumps vaccination rates (5). Several developed countries have added a second dose of the MMR vaccine to their national immunization programs for children aged 2–12 years to ensure durable immunity to mumps (46–48). Some reports have shown that, after the introduction of a second dose of the MMR vaccine in developed provinces in China, such as Beijing, Tianjin, and Shanghai, the annual incidence of mumps in these areas showed a decreasing trend (49, 52). Some studies have also shown that the antibody positivity rate increased to 98.00% after the second dose of the MMR vaccine, and the vaccine protection reached 83.00% (31). However, there is no relevant study on mumps vaccination in the Wuhan area. Since 2008, the MMR vaccination has been administered to children aged 18–24 months in Wuhan. High-risk groups may not have received the second dose of the MMR vaccine in a timely manner due to factors such as the remoteness of their areas, lack of medical resources, insufficient health awareness, and low vaccine coverage, which affects the vaccination rate. Therefore, children in Wuhan should receive the second dose of the MMR vaccine in a timely manner to reduce the incidence of mumps in high-risk age groups and to curb the spread of mumps among susceptible populations. The results of this study may provide evidence and clues for public health authorities to develop effective prevention and control measures and for further research on the risk factors related to mumps.

The present study has some limitations. Firstly, the optimal time period for implementing the intervention, i.e., the optimal time for vaccination, was suggested for the characteristics of mumps transmission in Wuhan, but the timing and effects of this intervention were not specifically evaluated; therefore, we need a transmission dynamics study for Wuhan after the intervention of the second dose of MMR vaccination.

## Conclusions

Many of the epidemiological features of mumps in Wuhan are consistent with those reported in local literature (50, 51). It was found that mumps in Wuhan has a peak period of nearly 5 years. The incidence was highest in children aged 5–10 years. It was highest from April to July and from November to January. There was no spatial correlation between the differences in the incidence rates and regions in Wuhan. The peak time of mumps transmission was 1–2 months earlier than the peak time of the outbreak. Modeling of mumps data in Wuhan from 2005 to 2018 revealed that vaccination played an important role in controlling the incidence of mumps. We recommend improving mumps vaccine coverage in Wuhan and implementing preventive and control measures, such as two vaccination doses for the 5 to 10-year-old population, before the peak of the transmission time to reduce the transmission of mumps in the population.

## Data Availability Statement

The data analyzed in this study is subject to the following licenses/restrictions: The datasets used and analyzed during the current study are available from Dr. Tianmu Chen (13698665@qq.com) on reasonable request. Requests to access these datasets should be directed to 13698665@qq.com.

## Author Contributions

TC and XY designed the research. YP, DK, BD, and WL collected the data. TY, YZ, CL, PL, ZZ, ZL, YW, JH, LL, JX, XL, JR, SL, MY, YP, DK, BD, WL, XY, and TC analyzed the data. YP, TY, YZ, XY, and TC wrote the manuscript. All authors contributed to the article and approved the submitted version.

## Conflict of Interest

The authors declare that the research was conducted in the absence of any commercial or financial relationships that could be construed as a potential conflict of interest.

## Publisher's Note

All claims expressed in this article are solely those of the authors and do not necessarily represent those of their affiliated organizations, or those of the publisher, the editors and the reviewers. Any product that may be evaluated in this article, or claim that may be made by its manufacturer, is not guaranteed or endorsed by the publisher.
